# Targeting Trichloroethylene-Induced Renal Endothelial Cell Injuries: A Role of Poly I:C in Amplification of HMGB1 Acetylation

**DOI:** 10.1155/jt/6652219

**Published:** 2025-08-13

**Authors:** Feng Wang, Yiting Hong, Jihong Gao, Ruixuan Cheng, Muyue Chen, Dandan Zang, Jiaxiang Zhang, Qixing Zhu

**Affiliations:** ^1^Department of Dermatology and Venereology, The Second Affiliated Hospital of Anhui Medical University, Hefei 230601, Anhui, China; ^2^Department of Occupational Health and Environmental Health, School of Public Health, Anhui Medical University, Hefei 230032, Anhui, China; ^3^Center for Scientific Research, Anhui Medical University, Hefei 230032, Anhui, China; ^4^Department of Dermatology and Venereology, The First Affiliated Hospital of Anhui Medical University, Hefei 230022, Anhui, China

**Keywords:** high-mobility group box protein 1, poly I:C, renal endothelial cell, toll-like receptor 3, trichloroethylene

## Abstract

Trichloroethylene hypersensitivity syndrome (THS), referred to as occupational medicamentosa-like dermatitis (OMDT) in China, typically manifests several days after exposure to trichloroethylene (TCE) in certain workers. Although our previous research has demonstrated that poly I:C exacerbates TCE-caused hepatitis in mice, the crucial role of poly I:C in THS renal injury remains largely unknown. In the current study, we focus on renal endothelial cell (EC) dysfunction after poly I:C treatment using a TCE-sensitized mouse model. Renal injury was evaluated in mice pretreated with poly I:C and compared to those without pretreatment, and the acetylation of high-mobility group box protein 1 (HMGB1) was also examined. Our results demonstrated that pretreatment with poly I:C worsened TCE-caused histological damage and functional impairment of mice kidneys. Notably, renal EC injury was identified as a key contributor to kidney damage, with poly I:C pretreatment amplifying these effects in the context of TCE sensitization. Moreover, our data showed that poly I:C, through its interaction with toll-like receptor 3 (TLR3), enhanced HMGB1 acetylation and subsequent release from renal ECs. Therefore, these key findings highlight a distinctive role of poly I:C in exacerbating TCE-caused renal EC injury. This study sheds new light on the complex interplay between viral mimicry and chemical sensitization, offering potential mechanistic explanations for THS pathogenesis. Our findings may help shape advanced strategies to prevent viral infections and address renal damage related to TCE exposure, with implications for clinical practice.

## 1. Introduction

Trichloroethylene (TCE), a widely used industrial halogenated hydrocarbon, is associated with various health issues [1, 2]. One of the most serious health consequences of TCE exposure is TCE hypersensitivity syndrome (THS), known as occupational medicamentosa-like dermatitis (OMDT) in China. This potentially fatal occupational condition arises due to insufficient protective measures during TCE exposure [3]. THS is characterized as not only by extensive skin lesions but also by damage to multiple organs, particularly the liver and kidney [3, 4]. Despite its significant contribution to global health issues, the underlying mechanisms driving TCE-induced kidney injury are still not well understood [5].

Although T cell-mediated delayed hypersensitivity reactions are often attributed to TCE-induced kidney injury [6], the exact mechanisms are still unclear. THS patients often exhibit symptoms resembling viral infections, such as fever, fatigue, and headache, prior to skin lesions and organ damage appear [7]. A Japanese research has found that about half of THS cases show elevated levels of herpes simplex virus DNA, with antiviral antibodies present in over 80% of cases [8]. Other investigations have suggested that human herpesvirus-6 and cytomegalovirus reactivation contribute to the initiation of THS [9].

Poly I:C, known as a synthetic dsRNA analog, is commonly employed to investigate innate immune responses [10, 11]. Once recognized by TLR3, a crucial receptor in viral infection, poly I:C triggers immune responses akin to those caused by dsRNA viruses [12]. In our previous studies, poly I:C has been utilized to mimic viral infections in mice, finding that pretreatment worsened hepatitis in TCE-sensitized animals [13]. However, the impact of viral infections on TCE-induced kidney injury has not been thoroughly investigated.

Renal endothelial cells (ECs), located along the glomerular capillaries, are crucial for kidney function maintenance. These cells participate in essential processes like blood filtration, blood flow, and pressure regulation and cellular communication in the renal microenvironment. Renal ECs are susceptible to damage during inflammation, fibrosis, and hypoxia, which can lead to kidney injury. Earlier research revealed interactions between ECs and podocytes via pathways like endothelin-1/endothelin receptor type A and angiopoietins/Tie-2, linked to TCE-induced glomerular injury [14]. However, the precise role of renal ECs in TCE-caused kidney damage requires further study. We used poly I:C to simulate viral infections in mice, offering a useful model to examine how viral infections might affect TCE-induced kidney injury.

## 2. Materials and Methods

### 2.1. Mice Groups and Treatments

**Figure 1:**
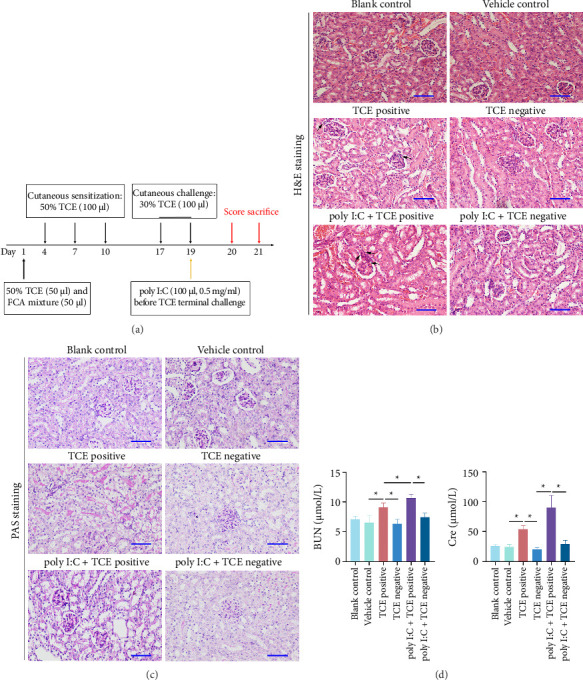
TCE sensitization caused kidney damage in mice. (a) Schematic representation of the murine TCE hypersensitivity model. (b) H&E staining of the kidney tissue (400× magnification). (c) PAS staining of the kidney tissue (400× magnification). Glomerular damage presented as hyperplasia of mesangial cells and disarray of cellular structure (as indicated by arrows) was noticeable in the TCE-positive group, with more severe pathology observed in the poly I:C + TCE group. (d) Assessment of renal function though the levels of BUN and Cre. Scale bars: 50 μm. ^∗^*p* < 0.05 indicates statistical significance.

Female BALB/c mice (6∼8 week old, *n* = 63) were sourced from the Center for Experimental Animals of Anhui Medical University and housed in a SPF facility with food and water provided *ad libitum*. The environmental conditions were set as follows: a 12 h light & dark cycle, 22.5 ± 0.5°C temperature, and 50 ± 5% humidity. After 7 days acclimatization, the TCE sensitization model was established following previously described protocols [13, 15]. Mice received a single intraperitoneal injection of poly I:C (100 μL of 0.5 mg/mL solution, InvivoGen, MA, USA) three hours before the challenge on Day 19 (Figure 1(a)). Sensitization was determined 24 h after the final challenge based on the presence of erythema and/or blisters.

Mice were then categorized into six groups based on treatment and skin response: Blank control group, vehicle control group, TCE-positive sensitization (TCE positive) group, TCE negative sensitization (TCE negative) group, poly I:C pretreatment + TCE positive sensitization (poly I:C + TCE positive) group, and poly I:C pretreatment + TCE negative sensitization (poly I:C + TCE negative) group. These protocols were reviewed and approved by the Experimental Animal Ethics Committee, Anhui Medical University (No. LLSC 20180310). All experiments were executed in alignment with national guidelines, the EU Directive 2010/63/EU, and ARRIVE guidelines.

### 2.2. Renal Damage Assessment

Fresh kidney tissue was collected and preserved in 4% paraformaldehyde for 48 h, and 5 μm thick sections were sliced for conduction with hematoxylin and eosin (H&E) staining and periodic acid-Schiff (PAS) staining. Mouse serum was collected, and blood urea nitrogen (BUN) and creatinine (Cre) levels were measured according to optical density (OD) values, measured using a plate under 640 nm for BUN and 546 nm wavelength.

### 2.3. Immunohistochemistry (IHC) Staining

Following the removal of paraffin and rehydration, renal sections were rinsed with PBS and endogenous peroxidase activity was blocked. For antigen retrieval, the sections were heated in a 0.01 M sodium citrate buffer solution. To prevent nonspecific antibody interactions, renal sections were treated with a 10% normal goat serum working solution and maintained at 37°C for half an hour. Then, the sections were incubated with specific primary antibodies targeting VCAM-1 (dilution 1:250, Abcam, UK), E-selectin (dilution 1:250, Abcam, UK), or ICAM-1 (dilution 1:300, Abcam, UK) overnight in a refrigerator. Subsequent steps included the application of biotin-conjugated secondary antibodies and streptavidin linked to horseradish peroxidase (supplied by ZSJQ-BIO, China). Finally, the presence of target proteins was detected using a 3, 3′-diaminobenzidine (DAB) detection kit.

### 2.4. Immunofluorescence (IF) Staining

Frozen mice kidney tissues preserved in OCT compound were sliced. These sections were subsequently fixed in acetone that had been precooled, for about 5 min. Post fixation, the sections were subjected to a blocking process with goat serum, which lasted at a temperature of 37°C for 2 h. Then, they were treated with antibodies specific to HMGB1 (dilution 1:250, Abcam, UK) and CD31 (dilution 1:250, Santa Cruz, US). Following this, the sections were exposed to secondary antibodies tagged with fluorescein for 2 h. Lastly, 4′,6-diamidino-2-phenylindole (DAPI, Merck, US) was applied to stain the nuclei.

### 2.5. Western Blot

Total proteins of mice kidneys were extracted, and the final concentration was standardized to 10 mg/mL. Aliquots of 10 μL were subjected to separation by SDS-PAGE and subsequently transferred onto a PVDF membrane (0.45 μm pore size, Millipore, USA). The membranes were then treated with BSA for blocking purposes over a period of 2 h. This was followed by incubation with specific primary antibodies targeting HMGB1 (dilution 1:1500, Abcam, UK), acetylated HMGB1 (dilution 1:1500, ABclonal, China), TLR3 (dilution 1:1500, ZEN-BIOSCIENCE, China), histone H3 (dilution 1:1500, Santa Cruz, USA), syndecan-1 (dilution 1:1500, Santa Cruz, USA), glypican-1 (dilution 1:1500, Santa Cruz, USA), and GAPDH (dilution 1:1500, Abcam, UK). Next, the membrane was exposed to a secondary antibody at a dilution of 1:10,000. Lastly, the protein bands were detected by a chemiluminescence imaging system.

### 2.6. Statistical Analysis

Data were presented as mean ± standard deviation (SD). One-way ANOVA, with Bonferroni's post-hoc test, was conducted for group comparisons using SPSS 13.0 software (Chicago, USA). A *p* value < 0.05 was defined as statistical significance.

## 3. Results

### 3.1. TCE Sensitization Caused Kidney Damage in Mice

The overall sensitization rate across all experimental mice (excluding blank control and vehicle control) was 33.33% (17 out of 51), based on skin reactions (Table 1). There is no statistical difference in sensitization rates between TCE treatment group (8 out of 25, 32%) and poly I:C + TCE treatment group (9 out of 26, 34.61%). To assess kidney injury, both histological changes and renal function were assessed. Representative kidney images are shown in Figure 1. Glomerular damage presented as hyperplasia of mesangial cells, and disarray of cellular structure was noticeable in the TCE-positive group. Notably, pretreatment with poly I:C seems to intensify these pathological alterations in mice from the poly I:C + TCE-positive group (Figures 1(b) and 1(c)). Consistent with histological findings, renal dysfunction, measured by elevated levels of BUN and Cre, was observed in mice from the TCE-positive group. Pretreatment with poly I:C further increased BUN and Cre levels in the poly I:C + TCE-positive group (Figure 1(d)), indicating that poly I:C may exacerbate TCE-induced kidney injury.

### 3.2. Renal EC Injury Aggravates Kidney Damage

**Figure 2:**
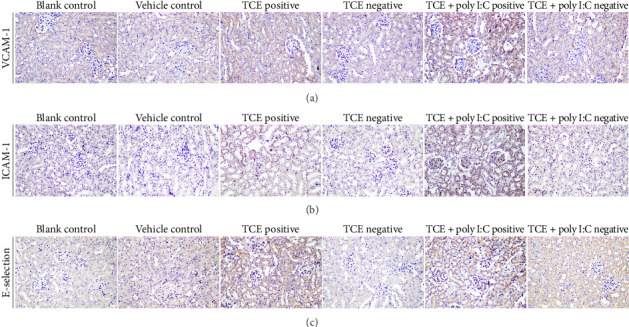
Elevated levels of ECs markers in TCE-sensitized mice. (a) VCAM-1 expression, (b) ICAM-1 expression, (c) E-selection expression in various groups (400× magnification).

**Figure 3:**
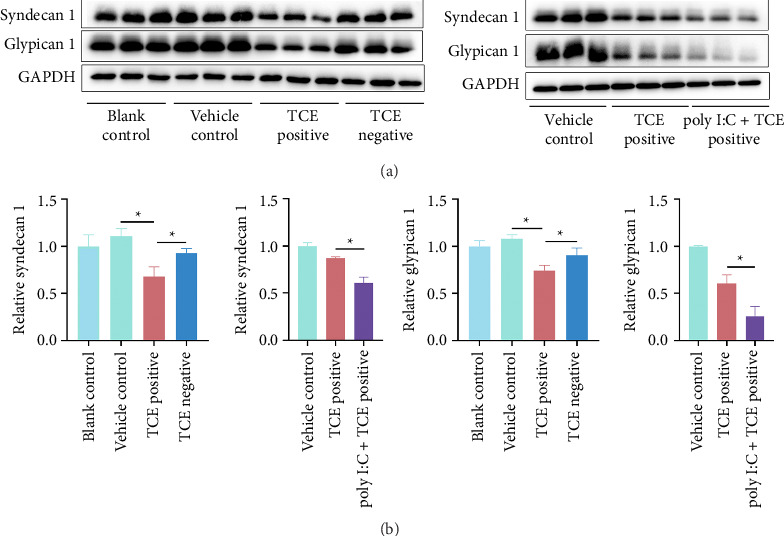
Elevated levels of ECs glycocalicin in TCE-sensitized mice. (a) Western blot images depicting protein levels of syndecan-1 and glypican-1. (b) Quantification of syndecan-1 and glypican-1 expression, normalized to GAPDH. ^∗^*p* < 0.05 indicates statistical significance.

Given the critical role of ECs in kidney disorders [16, 17], the current study focused on renal ECs. The EC activation in TCE-sensitized mice was confirmed with up-regulation of E-selectin, vascular cell adhesion molecule-1 (VCAM-1), and intercellular adhesion molecule-1 (ICAM-1) [18]. Elevated E-selectin, VCAM-1, and ICAM-1 levels were observed in the TCE-positive group compared to the TCE-negative group, with further increases noted in the poly I:C + TCE-positive group (Figure 2). Additionally, the expression of syndecan-1 and glypican-1, two essential components of the endothelial glycocalyx, was examined. Syndecan-1 and glypican-1 levels were reduced in the TCE-positive group relative to the TCE-negative group (*p* < 0.05) and were further diminished in the TCE + poly I:C-positive group (*p* < 0.05) (Figure 3, Supporting Figures show the original images of western blot (available here)). These results underscore the central involvement of ECs in TCE-caused kidney injury, with poly I:C exacerbating EC activation and glycocalyx degradation.

### 3.3. TLR3 Overexpression in Mouse Kidneys After Poly I:C Pretreatment

**Figure 4:**
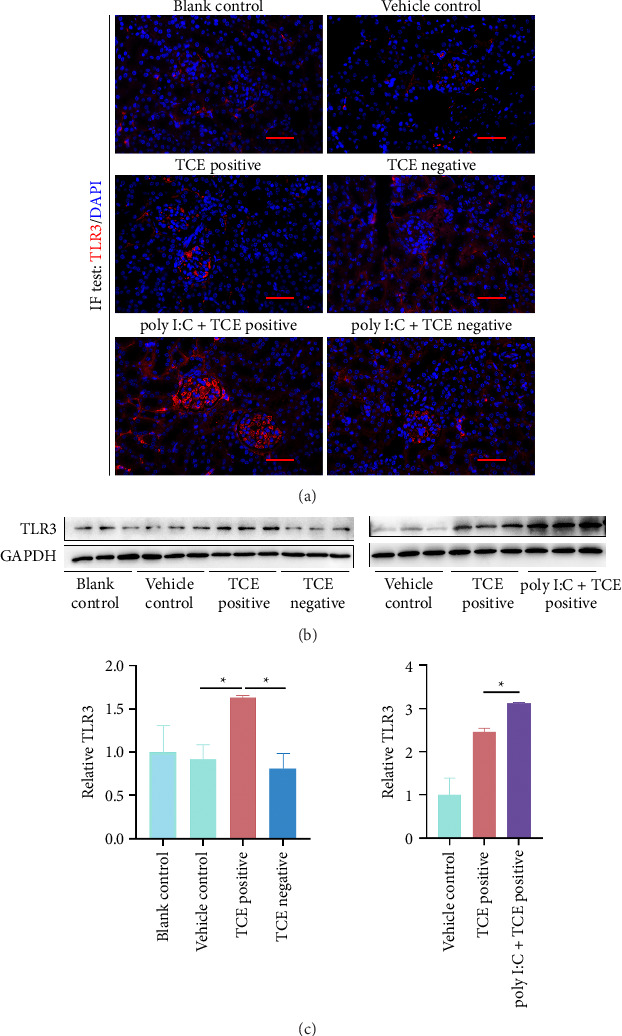
Elevated TLR3 expression in TCE-sensitized mice. (a) IF analysis of TLR3 expression in mouse kidneys (400× magnification). Scale bars: 50 μm. (b) Western blot bands showing TLR3 protein levels across different groups. (c) Quantification of TLR3 expression based on western blot results, normalized to a control. ^*∗*^*p* < 0.05 indicates statistical significance.

Considering the critical role of TLR3 in triggering innate immune responses during viral infections [19], we detected the expression of TLR3 using IF and western blotting in TCE-caused kidney injury. IF results showed higher TLR3 levels in the TCE-positive group vs. the control groups (both blank and vehicle control groups) or TCE-negative group, with further increases observed in the TCE + poly I:C-positive group (Figure 4(a)). Western blot analysis confirmed these findings, demonstrating increased TLR3 levels in TCE-sensitized mice, particularly in the TCE + poly I:C group (Figures 4(b) and 4(c), Supporting Figures show the original images of western blot). These findings suggest that poly I:C interacts with TLR3, potentially exacerbating kidney injuries during TCE sensitization.

### 3.4. Poly I:C Pretreatment Promoted HMGB1 Cytoplasmic Translocation

**Figure 5:**
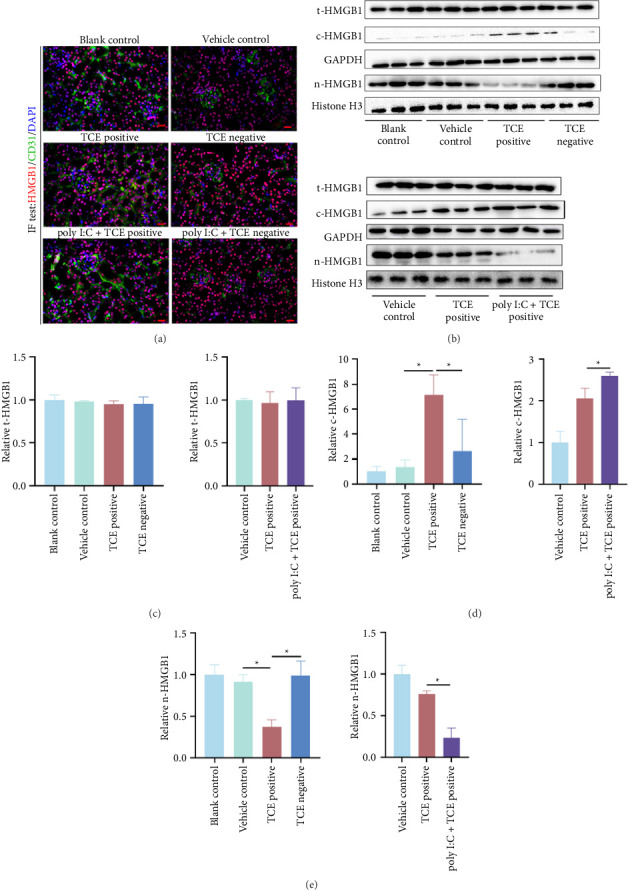
Poly I:C pretreatment facilitates HMGB1 translocation to the cytoplasm in mice. (a) Colocalization of HMGB1 and CD31 in mouse kidneys, visualized by immunofluorescence (400× magnification). Scale bars: 50 μm. (b) Western blot bands depicting total HMGB1 (t-HMGB1), cytoplasmic HMGB1 (c-HMGB1), and nuclear HMGB1 (n-HMGB1) protein levels. (c–e) Quantification of n-HMGB1, c-HMGB1, and t-HMGB1 expression, normalized to GAPDH or histone H3. ^∗^*p* < 0.05 indicates statistical significance.

As an alarmin in innate immunity, HMGB1 plays a critical role in viral replication and tissue damage [20, 21]. Upon stimulation, HMGB can translocate from nuclear to the cytoplasm and thereafter be released, acting as damage-associated molecular patterns (DAMPs). In this study, HMGB1 translocation was observed in the context of TCE-induced kidney injury. Using CD31 as a maker of renal EC, HMGB1 was localized to the nuclei of renal cells in blank control, vehicle control, and TCE-negative group (Figure 5(a)). Renal ECs were identified as a primary reservoir for HMGB1 storage. Analysis of nuclear HMGB1 (n-HMGB1), cytoplasmic HMGB1 (c-HMGB1), and total HMGB1 (t-HMGB1) levels revealed no significant changes in t-HMGB1 across groups (*p* > 0.05). However, c-HMGB1 levels were significantly upregulated in the TCE-positive group compared to the blank control or vehicle group and further increased in the poly I:C + TCE-positive group. Conversely, n-HMGB1 levels decreased in the TCE-positive group and were further reduced in the poly I:C + TCE-positive group (Figures 5(b), 5(c), 5(d), and 5(e), Supporting Figures show the original images of western blot). These findings suggest that poly I:C promotes HMGB1 translocation in renal ECs, potentially exacerbating kidney injury in TCE-sensitized mice.

### 3.5. Poly I:C Pretreatment Amplifies HMGB1 Acetylation in Murine Kidneys

**Figure 6:**
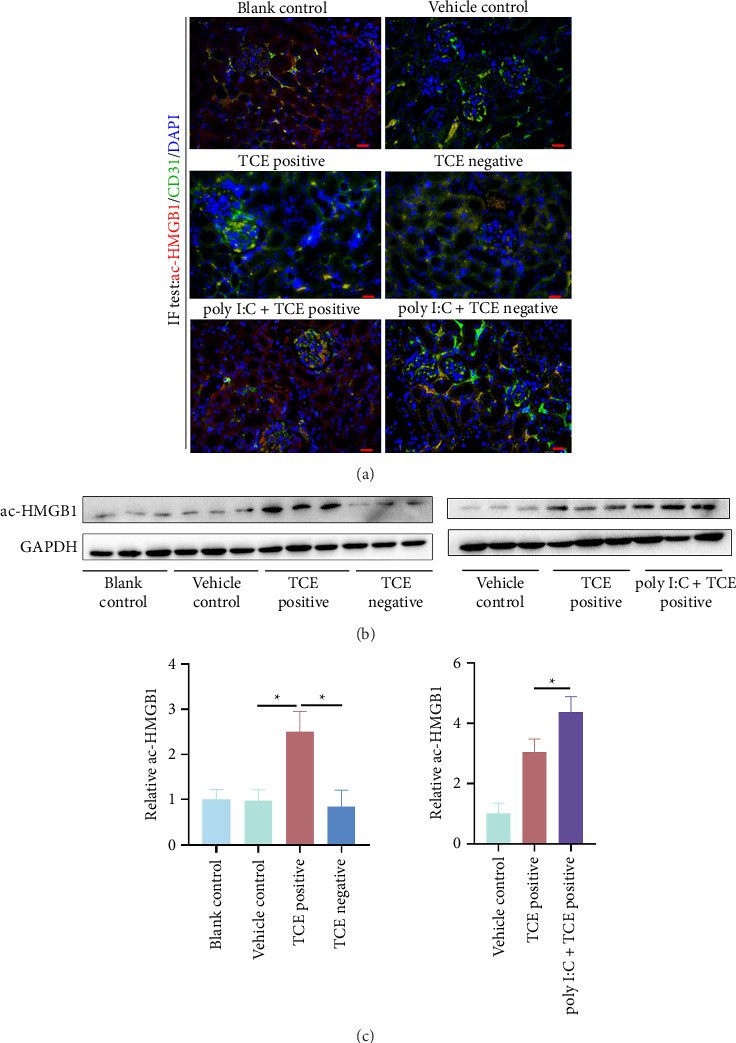
Poly I:C pretreatment enhances HMGB1 acetylation in mouse kidneys. (a) Colocalization of acetylated HMGB1 (ac-HMGB1) and CD31 in mouse kidneys, shown by immunofluorescence (400× magnification). Scale bars: 50 μm. (b) Western blot bands displaying acetylated HMGB1 (ac-HMGB1) expression across different groups. (c) Quantification of ac-HMGB1 levels, normalized to GAPDH. ^∗^*p* < 0.05 indicates statistical significance.

To investigate HMGB1 translocation, HMGB1 acetylation was assessed using IF and western blotting. IF analysis identified renal ECs as a major source of acetylated HMGB1 (ac-HMGB1), particularly in the glomerulus (Figure 6(a)). Western blot results showed increased ac-HMGB1 levels in the TCE-positive group compared to the TCE-negative group, with even greater increases observed in the poly I:C + TCE-positive group (*p* < 0.05) (Figures 6(b) and 6(c), Supporting figures show the original images of western blot). These findings indicate that HMGB1 acetylation may drive its cytoplasmic translocation in renal ECs, with poly I:C treatment further amplifying this process in mice.

## 4. Discussion

Exposure to TCE has been linked to kidney injury and related health complications [4, 22]. Although less frequent than hepatitis, kidney damage caused by TCE often correlates with a poor prognosis for patients with THS [23]. In prior research, we investigated the mechanisms underlying TCE-induced kidney injury, revealing that immune-mediated damage to renal cells contributes to both glomerular and tubular dysfunction [5, 22–26]. Notably, excessive complement activation, a key component of innate immunity, has been shown to impair podocytes and tubular epithelial cells, leading to renal damage [22, 25, 26]. However, the role of renal ECs in TCE-caused kidney injuries remains poorly understood. In this study, we assessed histopathological changes and EC activation markers, providing critical evidence of renal EC injury in TCE-exposed hosts.

Our findings also highlight the role of poly I:C in exacerbating renal EC injury in TCE-sensitized mice. Accumulating evidence suggests a connection between viral infections and various allergic disorders [27, 28]. In THS, clinical studies have documented flu-like symptoms, such as headache and fever, at the initial stage of the disease [7]. In particular, viral replication has been identified in THS patients [8]. Therefore, we pretreated mice with poly I:C as described in our previous work. Our results demonstrate that poly I:C pretreatment worsens TCE-caused renal damage in mice, which directly correlates with increased EC activation and injury. These findings underscore that viral factors might be involved in the initiation of TCE sensitization and associated kidney damage.

Given the role in viral infection surveillance, we investigated TLR3 (the receptor for poly I:C) in this study. TLR3 recognizes dsRNA, a byproduct of viral replication, and initiates innate immune responses to combat viral invaders [29]. While TLR3 has been well documented in the context of viral infections, its role in allergic diseases remains poorly understood. Previous research has implicated TLR3 in modulating specific phenotypes of allergic conditions such as asthma [30]. TLR3 activation works in orchestrating Th1 and Th2 immune responses, exacerbating allergic reactions [31]. In this study, we found altered TLR3 expression in the kidneys of TCE-treated mice, suggesting a role for TLR3 in amplifying TCE-induced allergic responses.

To better understand how poly I:C pretreatment exacerbates the effects of TCE sensitization, we investigated HMGB1 signaling in the kidneys of TCE-treated mice. HMGB1 is an alarmin molecule which activates innate immunity and plays a critical role in the regulation of allergic response [32]. Studies have shown that HMGB1 can influence CD4^+^ T cell differentiation, mediating inflammatory responses in conditions of allergic asthma [33]. Additionally, in vitro studies have demonstrated that HMGB1 upregulates adhesion molecules like ICAM-1 and P-selectin, leading to EC activation [34]. In this study, we found that poly I:C pretreatment enhanced HMGB1 acetylation, promoting its translocation in renal ECs from the nucleus to the cytoplasm. HMGB1 migration provides a plausible explanation for the exacerbation of renal EC injury by poly I:C pretreatment in TCE-sensitized mice.

Despite these insights, several limitations of this study should be acknowledged. First, the BALB/c mouse model for TCE sensitization differs from the classical guinea pig maximization test (GPMT) used for evaluating chemical sensitization. The mouse model was chosen due to the limited availability of reagents and antibodies for guinea pigs. Second, only female mice were used in this study, as no sex-based differences have been reported in TCE-sensitized workers. Female mice were selected because they typically exhibit more consistent and pronounced inflammatory responses, making them suitable for studying allergic diseases.

## 5. Conclusion

Our study highlights the role of poly I:C pretreatment in exacerbating TCE-induced kidney damage. We identified a novel mechanism by which poly I:C pretreatment enhances HMGB1 acetylation, leading to its cytoplasmic translocation and aggravating renal EC injury in TCE-sensitized mice [35]. These findings deepen our understanding of the interplay between viral infections and THS pathogenesis, emphasizing the importance of preventing viral infections in the clinical management of THS patients.

## Figures and Tables

**Table 1:** Mice groups and sensitization rate in the present study.

Groups	Mice (*n*)	Score			Sensitization (%)
		0	1	2	
Blank control	6	6	0	0	0
Vehicle control	6	6	0	0	0
TCE treatment	25	17	7	1	32
TCE positive	8	0	7	1	—
TCE negative	17	17	0	0	—
poly I:C + TCE treatment	26	17	8	1	34.61
poly I:C + TCE positive	9	0	8	1	—
poly I:C + TCE negative	17	17	0	0	—

## Data Availability

The data that support the findings of this study are available from the corresponding author upon reasonable request.

## Supporting Information

The original images of western blot are provided as Supporting Information.
