# Supplementary material for: Targeting Trichloroethylene-Induced Renal Endothelial Cell Injuries: A Role of Poly I:C in Amplification of HMGB1 Acetylation
Source: J Toxicol. 2025 Aug 13;2025:6652219. doi: 10.1155/jt/6652219 (PMC12367363; doi:10.1155/jt/6652219)

**Uncropped Western blot images including protein markers**

**Title: Targeting trichloroethylene-induced renal endothelial cell injuries: A role of poly I:C in amplification of HMGB1 acetylation**

**Figure 3A: Syndecan-1**


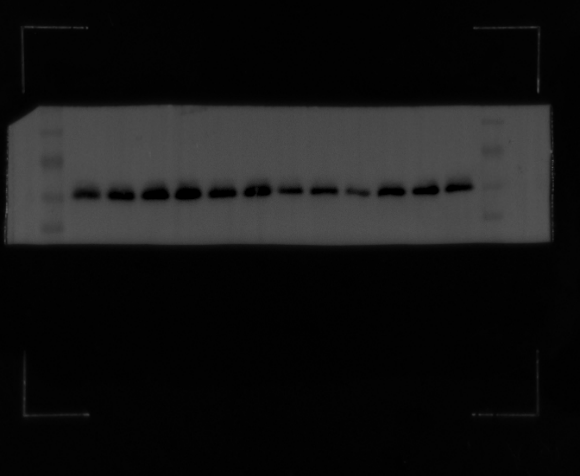

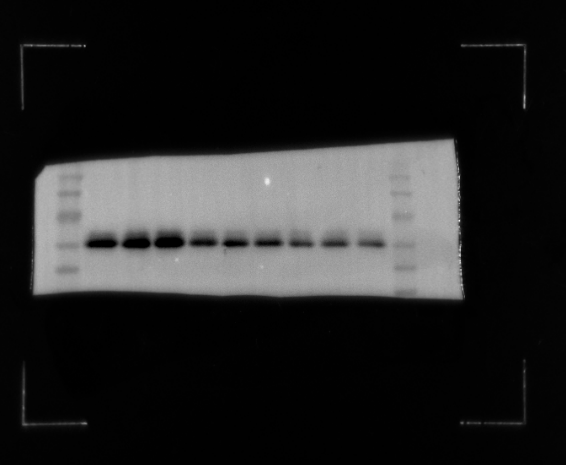


**Figure 3A: Glypican-1**


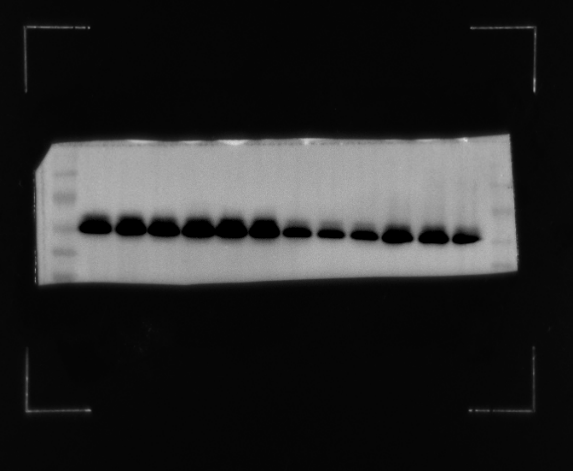

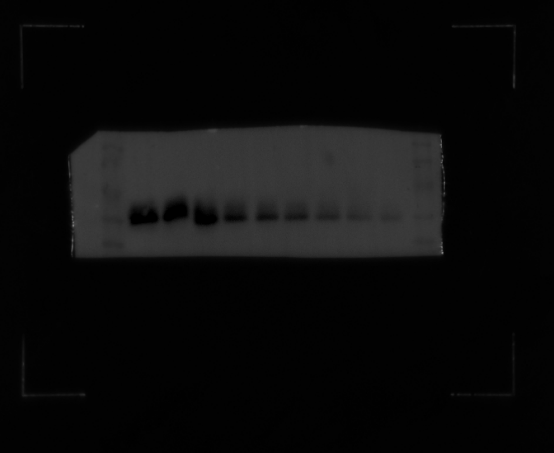


**Figure 3A: GAPDH**


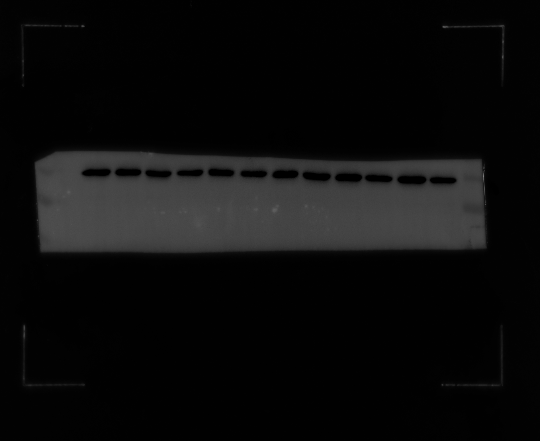

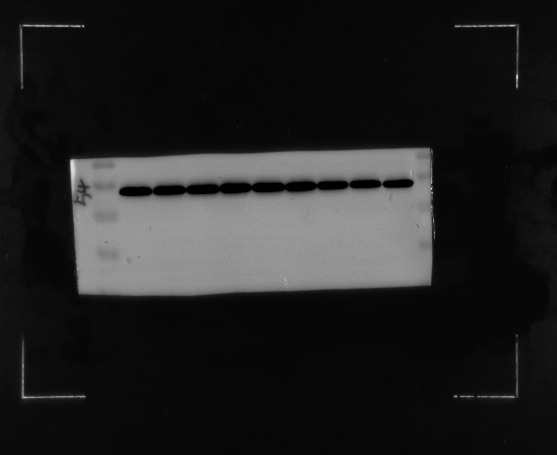


**Figure 4B: TLR3**


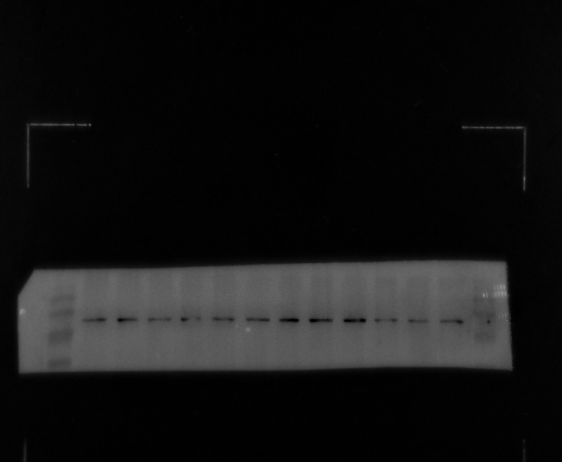

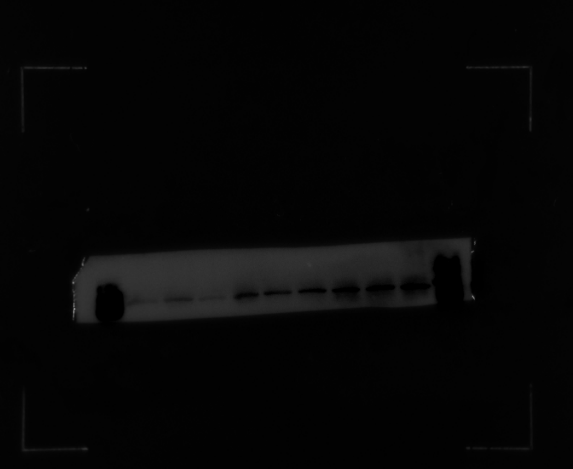


**Figure 4B: GAPDH**


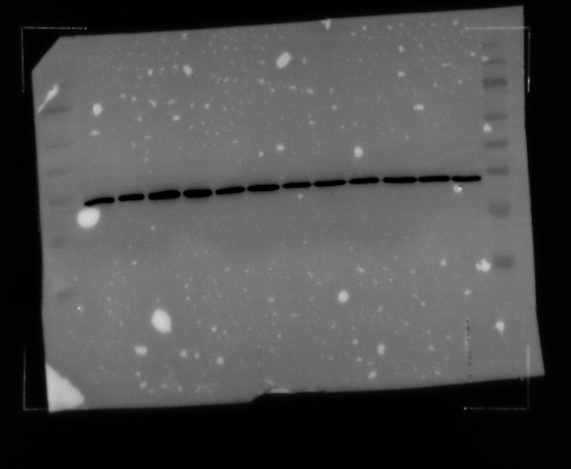

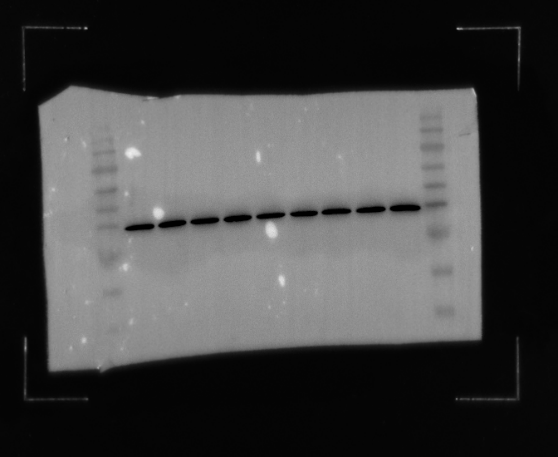


**Figure 5B: t-HMGB1**


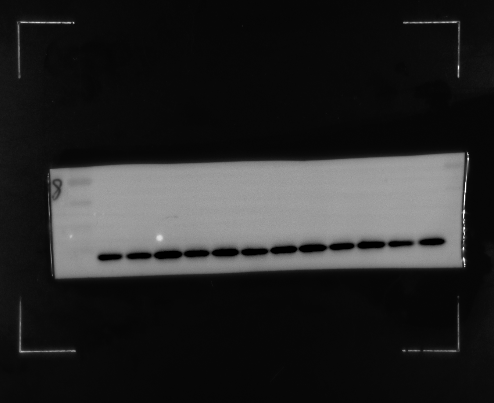

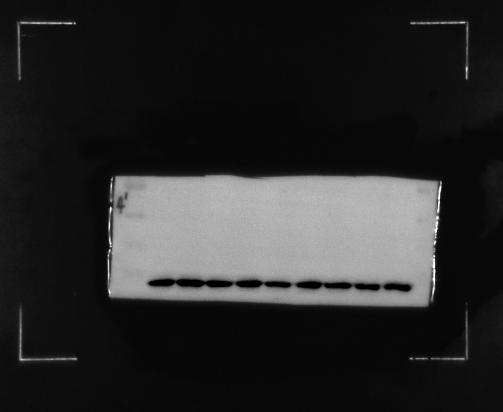


**Figure 5B: c-HMGB1**


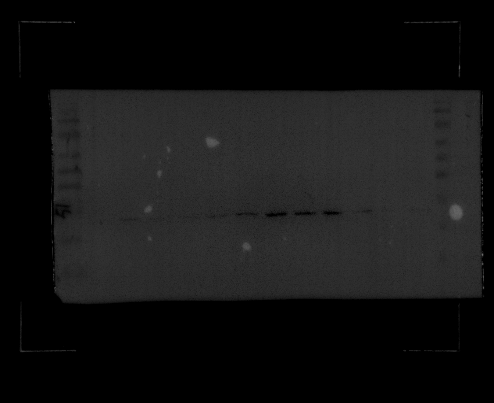

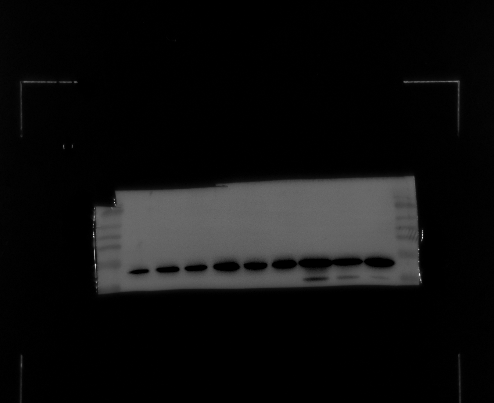


**Figure 5B: GAPDH**


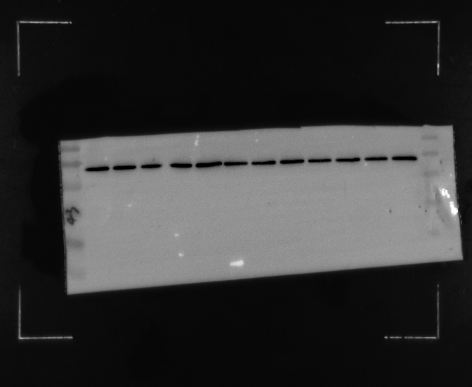

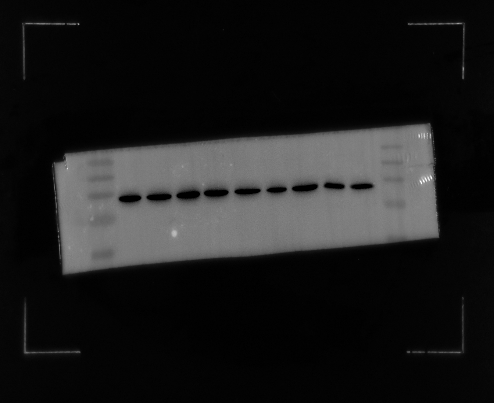


**Figure 5B: n-HMGB1**


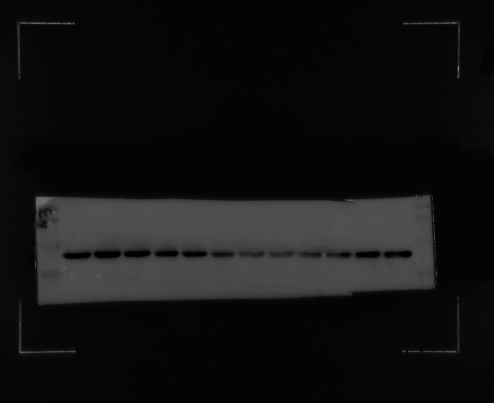

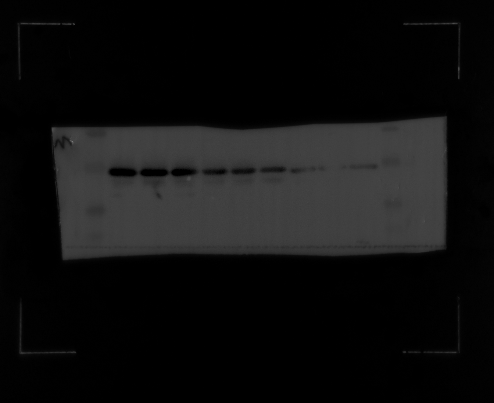


**Figure 5B: Histone H3**


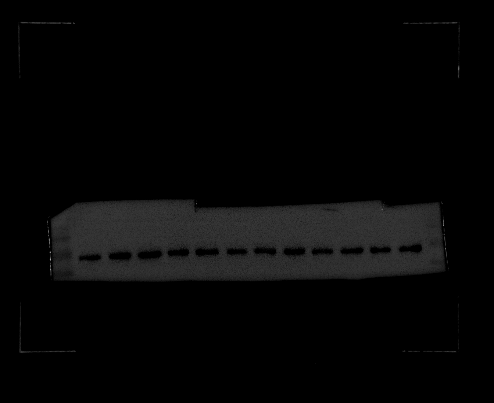

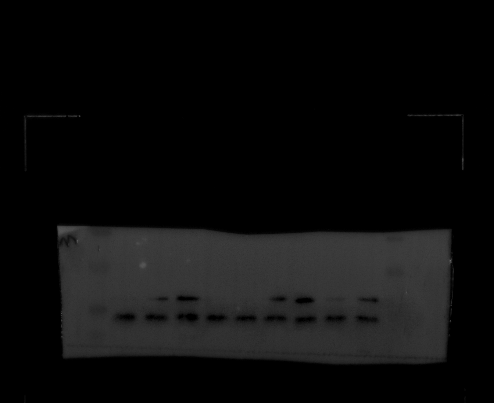


**Figure 6B: ac-HMGB1**


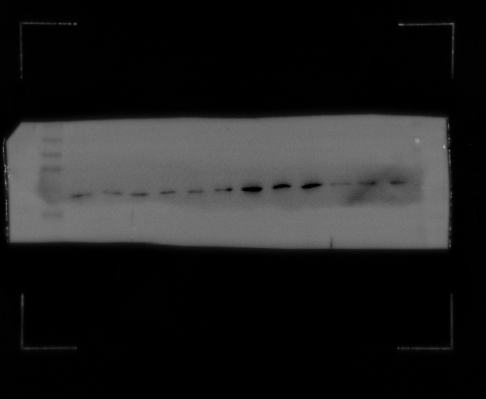

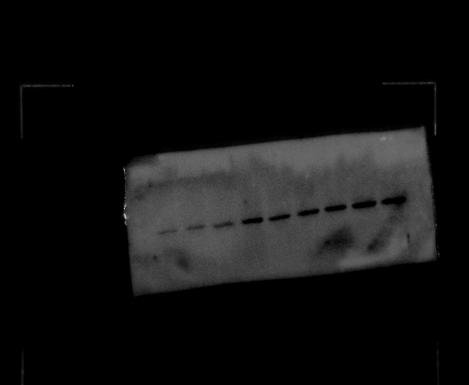


**Figure 6B: GAPDH**


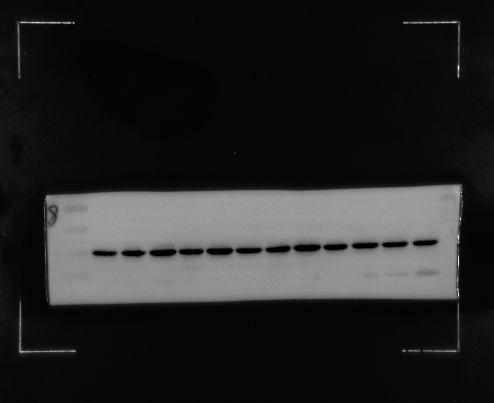

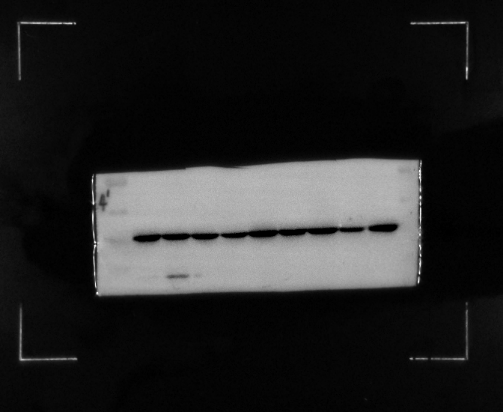

Supplement: Supporting Information — Additional supporting information can be found online in the Supporting Information section. [file 6652219.f1.docx]
